# Effect of synbiotic supplementation on children with atopic dermatitis: an observational prospective study

**DOI:** 10.1007/s00431-018-3253-4

**Published:** 2018-09-26

**Authors:** M. Dolores Ibáñez, Pablo Rodríguez del Río, Diego González-Segura Alsina, Vicenç Villegas Iglesias

**Affiliations:** 1 Allergy Department and Biomedical Research Foundation (FIB), Hospital Infantil Universitario Niño Jesús, Avda. Menéndez Pelayo 56, E-28009 Madrid, Spain; 2 Health Research Institute, IIS Princesa, Madrid, Spain; 3 Allergy National Network (ARADyAL), Institute Carlos III, Madrid, Spain; 4 Medical department, Chiesi Spain S.A.U., Barcelona, Spain

**Keywords:** Pediatric atopic dermatitis, Probiotics, Synbiotics, *Lactobacillus casei*, *Bifidobacterium lactis*, *Lactobacillus rhamnosus*, *Lactobacillus plantarum*

## Abstract

The objective of this observational single-cohort prospective study was to assess the effect of synbiotic supplementation for 8 weeks in children with atopic dermatitis (AD). The synbiotic product contained *Lactobacillus casei*, *Bifidobacterium lactis*, *Lactobacillus rhamnosus*, *Lactobacillus plantarum*, fructooligosaccharide, galactooligosaccharide, and biotin. Patients were examined at baseline and at 8 weeks. Effectiveness of treatment was assessed with the Scoring Atopic Dermatitis (SCORAD) index. A total of 320 children (mean age 5.1 years, range 0–12 years) were included. The mean (SD) SCORAD index decreased from 45.5 (15.5) at baseline to 19.4 (14.6) at the end of treatment (*P* < 0.001), VAS score for pruritus decreased from 5.7 (2.2) to 2.3 (2.2) (*P* < 0.001), and VAS score for sleep decreased from 3.1 (2.5) to 1.1 (1.8) (*P* < 0.001). Percentage of children with moderate-severe disease decreased from 92.4% at baseline to 28.1% at week 8. In the multiple linear regression analysis, higher baseline SCORAD index (OR 0.51; 95% CI 0.41–0.61) and higher adherence (OR 7.29; 95% CI 1.85–12.73) were significantly associated with greater decrease in SCORAD index.

*Conclusion*: Supplementation with the multistrain synbiotic product may improve AD in children.

**Table:** 

**What is known:** • *Pediatric atopic dermatitis (AD) is a common, troublesome condition with limited treatment options, which has been shown to be associated with dysbiosis in the intestinal microflora.* • *Results of controlled clinical trials (RCTs) on the effect of probiotics in children with AD have been disparate, although overall, the data favor probiotics over placebo, with multistrain supplements associated with better improvements in AD.*	
**What is new:** • *The results of this observational, prospective, open-label, single-cohort study on 320 children with AD younger than 12 years old suggest that supplementation with multistrain synbiotics (Lactobacillus casei, Bifidobacterium lactis, Lactobacillus rhamnosus, Lactobacillus plantarum, fructooligosaccharide, galactooligosaccharide, and biotin) helps to improve AD symptoms in children.* • *More than 80% of children experienced improvement in AD symptoms, as measured by Severity Scoring of Atopic Dermatitis (SCORAD) index and assessed by parents and physicians. The main predictive factors for improvement was adherence to synbiotic treatment and high baseline SCORE index; the change in SCORAD did not depend on age, gender, presence of concomitant treatment, duration, and type of AD (persistent vs with flares), other concomitant allergies or history of parental allergy.*

## Introduction

Atopic dermatitis (AD) is a common chronic, relapsing inflammatory allergic disease with highly pruritic skin lesions, particularly prevalent in children. It usually starts in the first 5 years of life and often has a profound negative effect on the quality of life of patients and their families [1]. AD affects up to 20% of children and 3% of adults, with the prevalence increasing globally [18].

The successful management of AD includes hydration, restoration of the skin barrier, control of skin inflammation, and treatment of secondary infections [10, 14]. Topical corticosteroids remain the first-line medical treatment for the control of symptoms, but relapses are common [20] and adverse effects limit their chronic use [11]. Calcineurin inhibitors are sometimes effective in reducing inflammation and help spare the use of topical steroids [5].

Synbiotics have been defined as combinations of pre- and probiotics with a synergistic action on human health [8]. Prebiotics are food components that induce the growth or activity of probiotics, which are living organisms that, when administered in adequate amounts (at least 10^9^ colony-forming units [CFU]), can be beneficial in the treatment of various conditions. Widely known probiotics such as *Bifidobacteria* and *Lactobacilli* have been identified as key components for proper immune system stimulation and homeostasis of the gastrointestinal tract microenvironment [7, 21]. Dysbiosis with increased levels of *Clostridium* and low levels of *Bifidobacterium* species in the intestinal microbiota has been observed in atopic children and was speculated to contribute to inflammation in AD [13]. Balancing the gut microflora may improve gut barrier function and reduce the production of proinflammatory cytokines. In addition, several in vitro and clinical studies showed that consumption of probiotics suppressed Th2 response and shifted Th1/Th2 balance towards Th1 response [16, 17]. Recently, modulation of microbiota to promote clinical improvement in pediatric patients with AD has been a focus of increasing interest. Meta-analyses of randomized controlled trials have shown that multistrain pro- and synbiotics are of benefit for the prevention and treatment of AD in children [2–4, 6].

The main objective of this prospective observational study was to assess the effect of a multistrain synbiotic supplement containing *Lactobacillus casei*, *Bifidobacterium lactis*, *Lactobacillus rhamnosus*, and *Lactobacillus plantarum* plus oligosaccharides and biotin for 8 weeks in children with AD. Treatment tolerance was also evaluated.

## Methods

### Study design and setting

This observational, prospective, multicenter study was carried out in the outpatient pediatric and allergology clinics throughout Spain in daily practice conditions. The study was conducted in accordance with the Declaration of Helsinki (7th revision), the Spanish regulations on observational studies (Order SAS 3470/2009) and Spanish personal data protection law (Law 15/1999). The study protocol was approved by the Ethics Committee of Hospital Infantil Universitario Niño Jesús, Madrid, Spain. Parents or legal representatives of all patients gave written informed consent before inclusion. All data were anonymized.

### Study population

The study population consisted of children < 12 years old, diagnosed with AD who presented with active eczema at the time of consultation at primary care centers. Exclusion criteria were allergy or intolerance to probiotics or excipients of the synbiotic product and presence of any severe disease other than AD as well as other dermatological diseases which could interfere with assessment of AD skin lesions. Patients were recruited between April 1 and August 31, 2016.

### Treatment and study procedures

The composition of the synbiotic product (1 g) was as follows: *L. casei* CBT LC5 4 × 10^9^ CFU, *B. lactis* CBT BL3 2 × 10^9^ CFU, *L. rhamnosus* CBT LR5 2 × 10^9^ CFU, *L. plantarum* CBT LP3 2 × 10^9^ CFU, biotin 7.5 mg, fructooligosaccharide 171.75 mg, and galactooligosaccharide 100 g (Produo® Derma, Chiesi España, S.A.U., L’Hospitalet de Llobregat, Barcelona, Spain). In this product, the probiotics were *L. casei*, *L. lactis*, *L. rhamnosus* and *L. plantarum*, while the prebiotics were fructooligosaccharide and galactooligosaccharide. A dose of 1 g (1 stick) twice a day was administered orally or dissolved in liquids or mixed with food. Treatment duration was 8 weeks**.**

The main study variable was changed in the Severity Scoring of Atopic Dermatitis (SCORAD) index [22]. Secondary variables were as follows: (1) demographic data (age, sex, parental education level); (2) profile of parental atopy (AD, food, drug, and respiratory allergy), living with pets, respiratory allergy, food allergy, drug allergy, duration of AD, age at onset, persistent (constant presence of symptoms) AD or flares (presence of symptoms intermittent with periods of remission); (3) concomitant medication; (4) VAS score for itch; (5) VAS score for sleep loss; (6) change in AD was qualitatively assessed by the physician in consultation with the parents as “very much improved,” “much improved,” “no change,” and “worse”; and (7) adverse events. Adherence to the synbiotic was calculated based on the patient’s diary and categorized as good (≥ 80%) or poor (< 80%). Patients were assessed at baseline and at the end of treatment (8 weeks).

### Statistical analysis

Based on the results of a randomized double-blind controlled trial carried out in a similar population of children with AD and in which a mean (standard deviation, SD) reduction of SCORAD of 39.2 (24.22) was found after 8 weeks of treatment with a synbiotic mixture [9], it was estimated that a sample of 867 patients would be needed to detect a mean change of 39.2 points in the SCORAD index, accepting a SD of 24.22 and a level of precision of 1.7 for a two-tailed analysis with an alpha error of 0.05. A lost to follow-up rate of 10% was assumed.

Analysis of the primary efficacy variable was performed in the intention-to-treat (ITT) data set, which included all patients treated with the synbiotic product independently of the level of adherence, and in the per-protocol (PP) data set, which included all patients who completed the study with a level of adherence of ≥ 80%. In the ITT analysis, the last observation carried forward was used for missing data. Categorical variables are expressed as frequencies and percentages, and continuous variables as mean and SD or median and interquartile range, with the corresponding 95% confidence interval (CI). The chi-square test, the Fisher’s exact probability test, or the McNemar’s test were used for the comparison of categorical variables, and the Student’s *t* test, the Mann-Whitney *U* test, or the Wilcoxon signed-rank test for the comparison of continuous variables according to normal or non-normal distribution of data. Agreement between variables was analyzed using kappa statistics. Variables independently associated with improvement of AD (expressed as difference between baseline and final SCORAD) were analyzed in a multiple linear regression model, in which all variables with a *P* value < 0.200 in bivariate analyses were included. Statistical significance was set at *P* < 0.05. The Statistical Package for the Social Sciences (SPSS Inc., Chicago, IL, USA) v22 was used for statistical analysis.

## Results

**Fig. 1:**
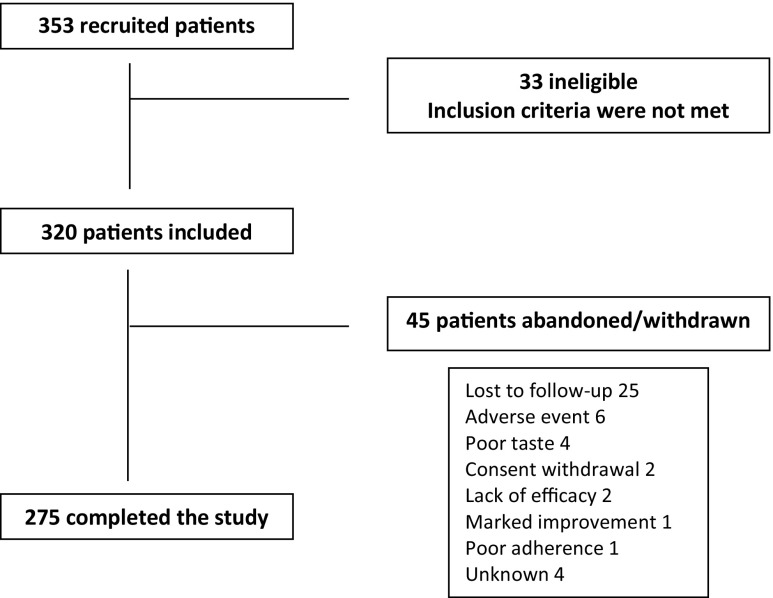
Flow diagram of participants in the study

The rate of recruitment was lower than expected, and a total of 353 patients were recruited within the foreseen period. Thirty-three (9.3%) patients did not meet the selection criteria and were excluded. Therefore, the study population included 320 patients, of which 275 completed the study. The flow diagram of participants is shown in Fig. 1.

Baseline characteristics are summarized in Table 1. Most (54.9%) patients were girls, and the median age was 4 years (range 3 months to 11.8 years). Concomitant respiratory allergy and food allergy were reported in 36.9% and 21.3% of patients, respectively; history of parental atopy was present in 70.9% of cases, most frequently AD or respiratory allergy. The median duration of AD was 3.4 years (IQR 1.7–6.2 years). Persistent AD was present in 52.1% of patients. Less than half of patients (43.4%) were receiving concomitant treatments, most frequently topical corticosteroids (21.3%), antihistamines (19.7%), or treatments for concomitant diseases (“Others,” Table 1). Thirty-three (11.1%) patients had changes in their concomitant treatment between the baseline and the follow-up visits.

**Table 1:** Baseline characteristics of 320 children with atopic dermatitis (AD)

Variables	Number (%)	Mean (SD)
Sex		
Girls	175 (54.9)	
Age, years		5.1 (3.1)
< 2 years of age	43 (13.4)	
≥ 2 and < 5 years of age	122 (38.1)	
≥ 5 years of age	155 (48.4)	
Parental history		
Education level, *n* = 304		
No studies	15 (4.9)	
Primary education	37 (12.2)	
Secondary education	109 (35.9)	
University degree	143 (47.0)	
Atopy, present	227 (70.9)	
AD	145 (45.3)	
Respiratory allergy	117 (36.6)	
Food allergy	36 (11.3)	
Drug allergy	25 (7.8)	
Living with pets	73 (22.8)	
Respiratory allergy, *n* = 309	114 (36.9)	
Food allergy, *n* = 301	64 (21.3)	
Drug allergy, *n* = 302	5 (1.7)	
Duration of AD, years		4.0 (3.0)
Type of disease, *n* = 292		
Persistent	152 (52.1)	
With flares	140 (47.9)	
Flares per month		1.9 (1.3)
Concomitant treatment*	139 (43.4)	
Systemic antibiotics	11 (3.4)	
Topical antibiotics	9 (2.8)	
Antihistamines	63 (19.7)	
Systemic corticosteroids	7 (2.2)	
Topical corticosteroids	68 (21.3)	
Topical immunosuppressants	12 (3.89)	
Other	71 (22.2)	

*A patient could have more than one concomitant treatment

**Fig. 2:**
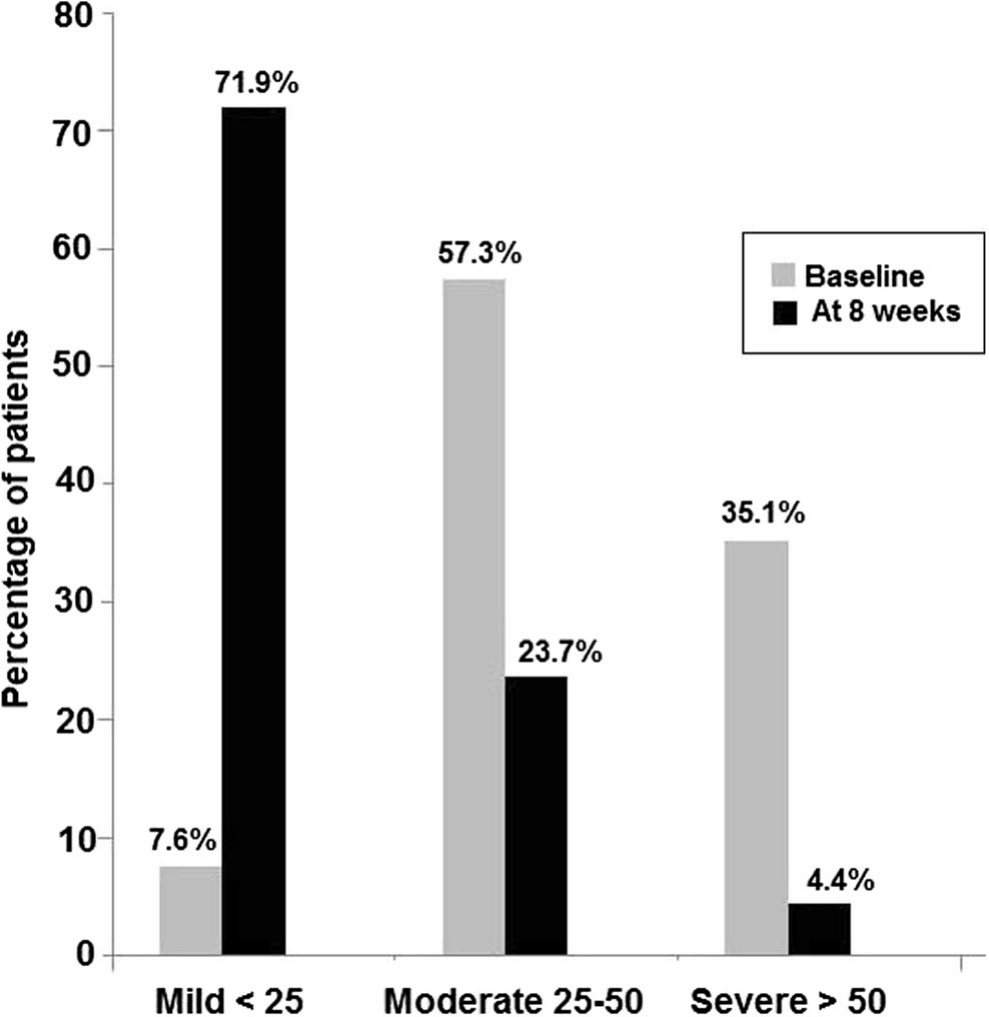
Changes in SCORAD scores between baseline and after 8 weeks of treatment with the synbiotic product. Differences for mild, moderate, and severe disease are statistically significant (*P* < 0.001)

At baseline, the mean (SD) SCORAD was 45.5 (15.5) (95% CI 43.8–47.3) (Table 2), and more than half of the patients (57.3%) had moderate disease (SCORAD 25–50) (Fig. 2). The intensity of the cutaneous symptoms was moderate in most patients. The mean VAS score for pruritus was 5.7 (2.2) (95% CI 5.5–5.9) and for sleep loss 3.1 (2.5) (95% CI 2.9–3.4). At the final visit (week 8), the mean SCORAD was 19.4 (14.6) (95% CI 17.7–21.2), cutaneous lesions had cleared or were of mild intensity in most patients, and the mean VAS score for pruritus was 2.3 (2.2) (95% CI 2.0–2.5) and for sleep loss 1.1 (1.8) (95% CI 0.9–1.3). Average (SD) intra-patient difference in SCORAD score between the basal and the follow-up visits was 27.0 (15.1) points (median 27.0; range − 6 to 79.3). All differences in SCORAD were statistically significant (*P* < 0.001) (Table 2). The number of patients with severe disease decreased from 35.1% at baseline to 4.4% at week 8 (Fig. 2). In the PP dataset, the mean SCORAD index decreased from 47.3 (15.5) (95% CI 45.3–49.3) at baseline to 19.2 (14.8) (95% CI 17.3–21.1) after 8 weeks of treatment (*P* < 0.001).

**Table 2:** Differences in results of the SCORAD index between baseline and the final visit after 8 weeks of treatment with the probiotic product

Variable	Baseline	Final visit (week 8)	*P* value
SCORAD score, mean (SD) (95% CI)	45.5 (15.5) (43.8–47.3)	19.4 (14.6) (17.7–21.2)	< 0.001*
Cutaneous symptoms, % of patients			
Erythema			< 0.001^†^
None	3.0	32.4	
Mild	23.3	52.7	
Moderate	58.5	12.2	
Severe	15.3	2.7	
Edema			< 0.001^†^
None	39.4	74.6	
Mild	35.5	18.1	
Moderate	21.1	6.0	
Severe	3.9	1.2	
Oozing/crusting			< 0.001^†^
None	36.2	75.1	
Mild	28.3	19.3	
Moderate	26.4	4.0	
Severe	9.1	1.6	
Excoriation			< 0.001^†^
None	18.1	59.9	
Mild	32.1	29.8	
Moderate	36.9	7.9	
Severe	12.9	2.4	
Skin thickening (lichenification)			< 0.001^†^
None	42.1	64.3	
Mild	27.4	27.0	
Moderate	22.6	7.9	
Severe	7.9	0.8	
Dryness			< 0.001^†^
None	1.7	15.6	
Mild	11.4	55.5	
Moderate	52.0	24.0	
Severe	34.9	4.9	
Pruritus, VAS score, mean (SD) (95% CI)	5.7 (2.2) (5.5–5.9)	2.3 (2.2) (2.0–2.5)	< 0.001*
Sleepiness, VAS score, mean (SD) (95% CI)	3.1 (2.5) (2.9–3.4)	1.1 (1.8) (0.9–1.3)	< 0.001*

*Wilcoxon signed-rank test; ^†^McNemar’s test

The percentage of physicians rating AD as “very much improved,” “much improved,” “no change,” and “worse” was 35.9%, 49.8%, 13.9%, and 0.3%, respectively. These percentages were similar to the parents’ opinion (38.9%, 42.7%, 17.4%, and 1.0%, respectively) (kappa 0.673). The kappa index for concordance between improvement/no improvement and 30% and 50% improvement of the SCORAD was 0.698 and 0.443 for physicians, and 0.643 and 0.520 for the parents.

Adherence to the synbiotic product was ≥ 80% in 89.7% of patients. The decrease in the SCORAD index was greater in patients with higher adherence (28.1 (14.9) points in adherent patients versus 18.0 (14.6) points in non-adherent patients (*P* = 0.004)).

In multiple linear regression analysis, the independent predictors of stronger improvement (that is, a greater decrease in the SCORAD index between baseline visit and week 8) were higher baseline SCORAD (OR 0.51, 95% CI 0.41–0.61; *P* = 0.0001) and adherence ≥ 80% (OR 7.29, 95% CI 1.85–12.73; *P* = 0.009) (Table 3).

**Table 3:** Summary of bivariate analyses and multiple regression analysis. The dependent variable was the intra-patient change in SCORAD index

Variable	Non-adjusted coefficients		*P*	Adjusted coefficients	*t*	95% CI for B	
	B	SD		Beta		Lower limit	Upper limit
Age	0.053	0.296	0.859	0.011	0.178	− 0.529	0.635
Sex	− 1.783	1.862	0.339	− 0.059	− 0.958	− 5.449	1.882
History of AD in parents	0.465	1.924	0.809	0.016	0.242	− 3.324	4.254
History of food allergy in parents	− 2.393	2.941	0.417	− 0.054	− 0.814	− 8.188	3.403
History of respiratory allergy in parents	2.926	1.981	*0.141*	0.094	1.477	− 0.976	6.827
History of drug allergy in parents	4.890	3.289	*0.138*	0.099	1.487	− 1.591	11.371
Living with pets	− 0.596	2.195	0.786	− 0.017	− 0.272	− 4.921	3.728
Parents’ education level	− 2.797	1.103	*0.012*	− 0.157	− 2.536	− 4.968	− 0.625
History of respiratory allergy in patient	1.853	1.944	0.341	0.059	0.953	− 1.974	5.681
History of food allergy in patient	4.464	2.270	*0.050*	0.123	1.967	− 0.006	8.935
History of drug allergy in patient	4.032	7.611	0.597	0.033	0.530	− 10.955	19.020
Duration of AD	0.401	0.319	0.210	0.082	1.256	− 0.228	1.031
Age at AD diagnosis	− 0.495	0.633	0.435	− 0.052	− 0.782	− 1.743	0.752
Type of AD (persistent vs with flares)	− 2.218	1.921	0.249	− 0.074	− 1.154	− 6.002	1.566
Basal SCORAD score	0.530	0.050	*< 0.001*	0.546	10.623	0.432	0.628
Season of start of treatment (summer vs spring)*	− 0.545	0.852	0.523	− 0.040	− 0.640	− 2.222	1.132
Adherence to probiotic (< 80% vs ≥ 80%)	10.059	3.247	*0.002*	0.190	3.098	3.664	16.454
Pre-existent concomitant treatment	0.766	2.884	0.791	0.024	0.265	− 4.943	6.474
De novo concomitant treatment	3.487	2.860	0.225	0.109	1.219	− 2.174	9.149
Any concomitant treatment	1.621	1.848	0.381	0.054	0.877	− 2.018	5.261
Best-fitting model							
Basal SCORAD score	0.508	0.050	*< 0.001*	0.528	10.103	0.409	0.608
Adherence to probiotic (< 80% vs ≥ 80%)	7.289	2.762	*0.009*	0.138	2.639	1.850	12.728

A total of 29 adverse events were recorded in 21 patients (Table 4). Of them, only two events of abdominal pain observed in the same patient were judged by the investigator to be potentially related to the product.

**Table 4:** Details of 29 adverse events registered in 21 patients

Adverse event	No. events	Causality with the synbiotic product
Cutaneous	10	
Worsening of dermatitis	2	Unrelated
Superinfection	2	Unrelated
Mollusculum contagiosum	2	Unrelated
Eczema	1	Unrelated
Erythema and scaling	1	Unrelated
Edema and pruritus	1	Unrelated
Pruritus and scaling	1	Unrelated
Respiratory	9	
Bronchospasm	4	Unrelated
Upper tract infection	4	Unrelated
Asthma attack	1	Unrelated
Gastrointestinal	7	
Acute gastroenteritis	4	Unrelated
Abdominal pain	2	Possibly related in 1
Vomiting	1	Unrelated
Neurological	2	
Irritability and insomnia	1	Unrelated
Nervousness, irritability, and insomnia	1	Unrelated

## Discussion

This study carried out in real-world practice shows that an oral supplementation with a synbiotic product with high levels of viable organisms for 8 weeks was effective in improving AD in children. The product included a mixture of four bacterial strains of commensal organisms (*L. casei*, *L. rhamnosus*, *L. plantarum*, and *B. lactis*), fructooligosaccharide, galactooligosaccharide, and biotin, the benefits of which in several immune-mediated and allergic diseases has been documented [15, 19, 23, 25]. Importantly, improvement was observed in long-standing AD (mean time from diagnosis of 4 years), both in persistent disease and in AD with flares, in patients with and without concomitant atopies, as well as in the presence or absence of underlying treatment for AD. These variables did not affect improvement of SCORAD index in bivariate analyses. Treatment was well tolerated, and of the 29 registered adverse events, only two events of abdominal pain were judged to be possibly related to the synbiotic product.

We used the SCORAD index, a valid and reliable tool, for the measurement of the main outcome of the study [22]. Statistically significant improvements in the total score as well as in the intensity of six cutaneous symptoms, pruritus, and sleepiness were found after 8 weeks of treatment. Also, the level of agreement of physicians and parents regarding amelioration of AD was substantial for a 30% decrease of the SCORAD score and moderate for a 50% decrease.

Conflicting data on the effect of probiotics on AD have been reported in literature, although in general, results favor probiotics over controls. A meta-analysis of 25 RCTs with 1599 AD patients found a greater improvement in SCORAD in the probiotics group compared to controls in children 1 to 18 years old (difference in change in SCORAD − 5.74 points vs control, 95% confidence interval − 7.27 to − 4.20), and in adults (difference in change in SCORAD − 8.26 points vs control, 95% confidence interval − 13.28 to − 3.25), although the effect was not proven in infants (< 1 year old) [12]. Similarly, a meta-analysis on RCT on synbiotics in children with AD (6 studies, 369 children) found a greater decrease in SCORAD (by 6.56 points) in synbiotic groups vs placebo (95% CI, − 11.43 to − 1.68) [6]. Heterogeneity was high in both meta-analyses, and in both cases, the authors found greater benefits in case of treatment with mixed bacterial species [6, 12].

Yesilova et al. [24] carried out a study in 40 children with AD who were randomized to supplementation with a probiotic mixture or placebo. Similarly, these patients were treated for 8 weeks with a high-dose (2 × 10^9^ CFU) multistrain probiotic complex containing *B. bifidum*, *L. acidophilus*, *L. casei*, and *L. salivarius*. The authors observed a significant decrease of SCORAD values at the end of treatment, with the magnitude of SCORAD reduction (mean change from baseline 23.0 points, compared to 12.8 points in placebo arm) comparable to that in our study (mean change from baseline 26.1 points). The probiotic supplement was also effective for reducing serum IL-5, IL-6, IFN-γ, and total serum IgE levels; unfortunately, the effect on IgE levels and proinflammatory cytokines was not examined in our study.

The improvement in SCORAD index and in all symptoms that was observed in our study was stronger than in most published controlled trials on probiotics in AD, which could be due to various reasons, including differences in study design, dose and strains of probiotics used, duration of intervention, characteristics of the study population, and the sample size. Since this was a single-arm, non-controlled study, we cannot rule out the placebo effect and/or improvement due to the natural course of the disease. It should be noted, however, that many participants had a long-standing AD (mean time since diagnosis 4 years), and about half had persistent disease, so it seems unlikely that all the improvement was due to the natural progression of the disease. Besides, since this was an observational study, there were no restrictions on concomitant medication, and 21% of study participants were receiving topical corticosteroids and 19% antihistamines. Overall, however, the percentage of patients receiving medical treatment for AD was lower than what could be expected in this population (most patients with moderate or even severe disease), which suggests that AD in our practice may be undertreated. Importantly, patients with and without concomitant medication showed a similar improvement in AD, suggesting that the observed changes could not be attributed exclusively to concomitant treatments. Furthermore, we found a significant association between the adherence to treatment with the multistrain high-dose synbiotic product and decrease of SCORAD index. Good adherence was the most important predictive factor of clinical improvement both in the bivariate and multivariate analyses, which further supports the idea that at least part of the observed improvement in AD was due to the synbiotic treatment.

One possible limitation affecting the validity of the results was that the participant rate was lower than the projected sample size. The observational character of the study allowed including a varied sample of patients, and the multivariate analysis allowed assessing the effectiveness of treatment in relation to patient’s characteristics. Well-designed RCTs should be conducted to elucidate the effectiveness of synbiotics in AD treatment. Including more representative real-world patient samples and complementing RCT with data from observational studies should help physicians treating children with AD to have appropriate evidence on which to base their clinical decisions in daily practice.

In conclusion, our results indicate that supplementation with multistrain high-dose synbiotics (*L. casei*, *L. rhamnosus*, *L. plantarum*, and *B. lactis*, combined with fructooligosaccharides, galactooligosaccharides, and biotin) improves AD in children, whereas the tolerability and safety profile are very good. Further studies, including RTCs, are needed to add evidence on the benefits of synbiotics in the treatment of pediatric AD in real-life clinical practice.

## Abbreviations

AD: Atopic dermatitis
CFU: Colony-forming units
CI: Confidence interval
ITT: Intention to treat
OR: Odds ratio
PP: Per protocol
RCT: Randomized controlled trials
SCORAD: Severity Scoring of Atopic Dermatitis
SD: Standard deviation
VAS: Visual analogue scale
